# Integrated Production of Microalgal Oil from *Neochloris oleoabundans* and Its Enzymatic Conversion into Mono- and Diacylglycerols

**DOI:** 10.3390/foods15132333

**Published:** 2026-07-01

**Authors:** Raphael Sena, Daniel Kurpan, Elisa d’Avila Costa Cavalcanti, Denise Maria Guimarães Freire, Anita Ferreira do Valle

**Affiliations:** 1 Department of Biochemistry, Federal University of Rio de Janeiro, Av. Athos da Silveira Ramos 149, Rio de Janeiro 21941-909, Brazil; o.senaraphael@gmail.com (R.S.);; 2 Food Microbial Systems, Agroscope, Schwarzenburgstrasse 161, 3003 Bern, Switzerland

**Keywords:** *Neochloris oleoabundans*, monoacylglycerol, diacylglycerol, enzymatic production

## Abstract

Microalgal lipids are promising sustainable feedstocks for high-value functional ingredients. However, the influence of cultivation-driven lipid composition on enzymatic conversion remains poorly understood. This study integrated cultivation strategy and enzymatic upgrading to tailor *Neochloris oleoabundans* lipids for mono- and diacylglycerol (MAG and DAG) production. Heterotrophic cultivation achieved a maximum dry biomass concentration of 2.78 ± 0.14 g L^−1^, whereas autotrophic cultivation reached 0.39 ± 0.01 g L^−1^, confirming the superior biomass productivity of heterotrophic metabolism. Lipid fractions obtained under both trophic conditions were characterized and subjected to glycerolysis catalyzed by Novozym 435 under a 5:1 glycerol-to-oil ratio for 16 h. Heterotrophic oils, characterized by triacylglycerol-rich and low-free fatty acid (FFA) profiles, achieved higher MAG + DAG conversion (45%), while autotrophic oils reached 43% conversion despite elevated FFAs and polar lipids. The presence of FFAs, pigments, and phospholipids in non-refined microalgal oils influenced catalytic behavior, reducing conversion efficiency and favoring competing esterification and hydrolysis pathways. These findings demonstrate that substrate purity, acylglycerol distribution, and cultivation-specific lipid architecture strongly affect lipase performance, highlighting oil refining and cultivation optimization as key strategies for improving sustainable MAG and DAG production.

## 1. Introduction

Global population is projected to exceed 9.8 billion by 2050, placing unprecedented pressure on agricultural systems and intensifying the demand for sustainable food production strategies [1]. Conventional vegetable oil crops such as soybean, palm, and canola remain central to global food and industrial sectors. However, their expansion is increasingly constrained by deforestation, biodiversity loss, high freshwater requirements, and soil degradation [2,3]. These limitations highlight the urgent need for alternative lipid sources capable of meeting growing demand while minimizing environmental impacts.

In this context, single-cell oils (SCOs) produced by microorganisms have emerged as promising alternatives due to their high lipid productivity, compositional flexibility, and favorable biochemical profiles. Among microbial platforms, microalgae are particularly attractive because of their rapid growth, capacity to accumulate substantial lipid fractions, and ability to be cultivated on non-arable land using saline water or wastewater, thereby minimizing competition with conventional agriculture [4,5,6]. These characteristics align microalgal lipid production with circular bioeconomy principles and long-term food security strategies.

Beyond serving as sustainable oil feedstocks, microalgal lipids can be converted into high-value functional ingredients. As the focus of this study, the transformation of microalgal oils into monoacylglycerols (MAGs) and diacylglycerols (DAGs) represents a promising route to meet the growing demand for emulsifiers with enhanced nutritional and functional properties. Oils rich in polyunsaturated fatty acids (PUFAs), including omega-3 and omega-6 fatty acids, are especially attractive substrates for MAG and DAG synthesis, yielding emulsifiers that not only stabilize emulsions and improve lipid dispersion but also contribute to improved nutritional quality and health-promoting attributes [7]. Moreover, the use of microalgae-derived emulsifiers offers advantages related to supply-chain resilience, traceability, and reduced greenhouse gas emissions compared with conventional plant-based lipid sources [8,9].

To enable efficient conversion of microalgal oils into structured emulsifiers, enzymatic catalysis has emerged as a preferred alternative to conventional chemical routes. While chemical glycerolysis and interesterification processes, typically conducted at 200–260 °C using alkaline catalysts, remain economically attractive, they often generate unwanted by-products, promote thermal degradation of PUFAs, and result in darker, less stable emulsifier mixtures [10]. In contrast, lipase-catalyzed reactions operate under mild conditions (40–70 °C), minimize side reactions, preserve thermolabile fatty acids, and enable regioselective control over product distribution, yielding MAG and DAG with higher purity and superior sensory and functional quality [11]. These advantages make enzymatic routes particularly suitable for high-value applications in food, cosmetic, and pharmaceutical formulations.

In this context, the chlorophyte *Neochloris oleoabundans* UTEX 1185, has emerged as a highly promising microalgal platform for SCO production. Originally isolated from the Rub’ al-Khali desert [12], this species exhibits robust growth and tolerance to freshwater, seawater, and wastewater environments, along with a structurally complex cell wall composed of carbohydrates and algaenan that confers enhanced environmental resilience [13]. Its lipid content typically reaches approximately 37% of dry biomass, with up to 58% of this fraction occurring as triacylglycerols (TAGs), making it particularly suitable as a feedstock for downstream lipid transformations [14]. Lipid accumulation can be further enhanced through cultivation strategies such as nitrogen deprivation or supplementation with humic substances, with reports of lipid contents approaching 49% under optimized conditions [15,16].

Despite these advantages, the direct application of crude microalgal oils remains limited by the presence of pigments, phospholipids, and oxidation-prone compounds, which necessitate refining prior to catalytic upgrading [17,18]. Furthermore, critical knowledge gaps remain regarding how cultivation conditions influence fatty acid profiles in *N. oleoabundans* oils and how these compositional variations affect enzymatic performance and selectivity during MAG and DAG synthesis. Addressing these gaps is essential for establishing integrated biorefinery platforms capable of converting microalgal biomass into high-value, food-grade functional lipids.

Therefore, this study proposes a process-oriented approach in which microalgal cultivation is strategically used to tailor lipid composition toward enhanced enzymatic conversion into mono- and diacylglycerols. By integrating cultivation conditions, lipid extraction, and enzymatic glycerolysis, this work aims to establish a direct link between upstream metabolic programming and downstream catalytic performance.

Overall, the integration of microalgal SCO production with enzymatic conversion into MAGs and DAGs represents a compelling strategy for developing next-generation food emulsifiers that combine technological performance, nutritional enhancement, and environmental sustainability. In this context, the cultivation and lipid upgrading of *N. oleoabundans* provides a renewable and scalable platform to support resilient, low-impact, and health-promoting food systems, in alignment with key United Nations Sustainable Development Goals (SDGs), including SDG 2 (Zero Hunger), SDG 12 (Responsible Consumption and Production), and SDG 13 (Climate Action) [1].

## 2. Materials and Methods

### 2.1. Cultivation and Experimental Design Parameters

Control cells were cultivated in Bold’s Basal Medium (BBM) as described by Andersen [19], with modifications to the parameters to promote lipid accumulation. These included the modulation of light intensity, sodium bicarbonate, glucose, glycerol, dipotassium phosphate, sodium chloride for salinity, sodium acetate, and sodium nitrate in microalgae cultures [20,21,22,23]. The cultures were maintained in 500-mL Erlenmeyer flasks on a bench-top shaker (New Brunswick C25KC, Edison, NJ, USA), with a 12/12 h photoperiod and a temperature of 30 °C for 15 days. A Plackett–Burman design was applied for parameter screening (Table 1), followed by a central composite rotatable design (CCRD) based on the Plackett-Burman results to optimize the cultivation conditions. The experimental matrices for autotrophic cultivation are presented in Table 2, while those for heterotrophic cultivation are presented in Table 3.

### 2.2. Nile Red Staining

To identify triglycerides in the microalgae, the fluorescent dye Nile Red was prepared in an acetone solution at a final concentration of 0.5 μg mL^−1^. The microalgae cultures were sampled every 2 days, with aliquots added to black, flat-bottomed 96-well plates [24]. Staining was performed by incubating samples with Nile Red for 10 min at 40 °C. Nile Red specifically binds to neutral lipids, enabling detection using a SpectraMax M2 plate reader (San Jose, CA, USA) with an excitation wavelength of 485 nm and an emission wavelength of 595 nm.

### 2.3. Lipid Extraction

For lipid extraction, a method was employed involving biomass previously dried at 30 °C for 24 h The extraction process began by adding methanol to the biomass in Falcon tubes containing dry microalgal biomass and glass beads at a 1:10 (w/w) ratio. The mixture was agitated for 30 min with 5-min intervals. Subsequently, hexane was added according to the desired final mixture (1:1 or 5:1), followed by 2 additional minutes of agitation. The mixture was then centrifuged at 5000× *g* for 4 min, and the oil was recovered from the supernatant and concentrated using a rotary evaporator (Büchi R-210, Flawil, Switzerland). These methods were compared with a well-established method used for microalgal lipid extraction previously described by Bligh and Dyer [25]. Lipid extraction was determined gravimetrically on a % dry biomass basis.

### 2.4. Determination of Fatty Acid Methyl Esters (FAME) Content

The determination of fatty acid methyl esters (FAMEs) was carried out following a derivatization process based on Cavalcanti-Oliveira et al. [26] using a GC-2030 gas chromatograph (Shimadzu Co., Kyoto, Japan) equipped with a flame ionization detector (FID; Shimadzu Co., Kyoto, Japan) and an Omegawax capillary column (30 m × 0.25 mm × 0.25 μL). The detector and injector were set at 250 °C and 260 °C, respectively. The oven program was set to 125 °C for 2 min, followed by heating at 10 °C min^−1^ to 240 °C, which was held for 5 min. Hydrogen was used as the carrier gas at a flow rate of 1.6 mL min^−1^. Sample aliquots of 20 μL were diluted, and 1 μL was injected with a 1:20 split ratio. Ester content was quantified using the internal standard peak area.

### 2.5. Enzymatic Production of Mono- and Diglycerides

Mono- and diglycerides (MAGs and DAGs) were synthesized via enzymatic glycerolysis. Reactions were performed using microalgae oil, soybean oil, or free fatty acids (FFAs) as substrates. Each substrate was mixed with free glycerol at a 1:5 molar ratio (oil:glycerol). The reaction mixtures were prepared in open glass vials to allow moisture release. Novozym 435 was added at 5% (w/w) relative to the total mass of substrates. The vials were incubated at 50 °C under constant agitation for 16 h. After incubation, the enzyme was removed by filtration.

### 2.6. Determination of Mono-, Di-, and Triglycerides (MAG, DAG, and TAG)

The quantification of residual mono-, di-, and triglycerides in the FAME samples was performed according to the EN 14105 standard [27]. For derivatization, 100 mg samples were mixed with N-methyl-N-trimethylsilyltrifluoroacetamide (MSTFA) in the presence of pyridine, promoting the silylation of glycerol, MAG, and DAG into volatile derivatives suitable for gas chromatographic analysis. The reaction mixture was incubated at 60 °C for 15 min, ensuring complete derivatization, and subsequently injected into a gas chromatograph equipped with an on-column injector, a polar capillary column as specified in the standard, and a flame ionization detector (FID).

Quantification was carried out using the designated internal standards: mono C19 for monoglycerides, di C38 for diglycerides, and tri C57 for triglycerides, along with butanetriol for free glycerol. Individual calibration curves were constructed for each analyte class. Concentrations of MAG, DAG, and TAG were expressed as % (w/w), calculated from the peak area ratios between analytes and their respective internal standards.

### 2.7. Statistical Analysis

Plackett-Burman designs and CCRD were carried out in Statistica 7.0 using the results matrix of the experimental design described in Section 2.1.

The reported data corresponded to the mean and standard deviation of at least three biologically independent replicates (*n* ≥ 3). The effects of categorical factors on results were analyzed by Student’s *t*-test for the comparison of two datasets (e.g., heterotrophic vs. autotrophic) or by one-way analysis of variance (ANOVA) for more than two datasets (e.g., lipid extraction using four different extraction methods). Tukey’s honest significant difference test was used to determine whether there were significant differences between each individual dataset. All statistical analyses were conducted with a confidence level of 95% (α = 0.05).

## 3. Results and Discussion

### 3.1. Selection of Cultivation Parameters

**Figure 1:**
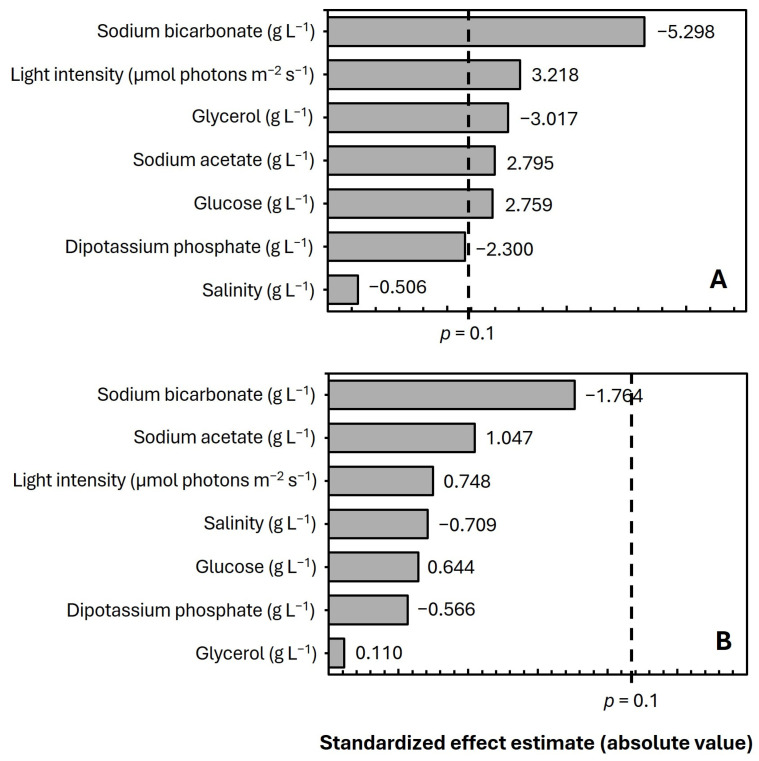
Pareto charts of standardized effects from the Plackett–Burman design: biomass concentration (g·L^−1^; (**A**)) and lipid content (g·L^−1^; (**B**)), highlighting the most influential variables on each response.

A Plackett–Burman design was initially applied under mixotrophic conditions to screen seven cultivation parameters and identify variables influencing biomass formation and lipid accumulation. Since an increased C/N ratio typically achieved through nitrogen limitation is a well-established trigger for lipid accumulation in microalgae, this parameter was intentionally excluded from the screening stage [23,28,29]. Regarding biomass production, the Pareto charts (Figure 1) indicated that sodium bicarbonate exerted a strong negative effect within the tested concentration range, followed by dipotassium phosphate. Salinity was the only parameter that did not reach statistical significance (α = 0.1). Nonetheless, previous studies have shown that salinity can stimulate both growth and lipid accumulation in *N. oleoabundans* [30] justifying its inclusion in subsequent analyses.

Although the factors evaluated in this screening are widely recognized for promoting lipid accumulation in microalgae, none exhibited statistical significance for lipid production in this initial design. However, observed response trends suggested that the tested ranges were not optimal for detecting statistically significant effects. Therefore, all variables were reassessed and their concentration intervals adjusted for subsequent evaluation in the CCRD. However, sodium acetate and dipotassium phosphate were excluded from the next experimental stage, as they did not exhibit statistically relevant effects on biomass or lipid responses in preliminary analyses, allowing the optimization effort to focus on the most influential parameters.

The multifactorial evaluation performed using two CCRDs (one under photoautotrophic conditions and the other under heterotrophic metabolism) revealed that biomass formation and lipid accumulation are governed by distinct environmental and nutritional drivers in *N. oleoabundans*. Overall, the results demonstrate that growth and lipid biosynthesis are regulated by fundamentally different factors and nonlinear interactions. Under photoautotrophic conditions, the quadratic models for biomass and lipid production were significant (*p* < 0.05) and showed a coefficient of determination (R^2^) of 0.949 and 0.968, respectively (Table S1; Figure S1).

*y*_(biomass)_ = 1.530279 − 0.001312 *x*_1_ + 0.0000002 *x*_1_^2^ − 0.117755 *x*_2_ + 0.002333 *x*_2_^2^ + 0.000111 *x*_1_*x*_2_

*y*_(lipid)_ = 0.203134 + 0.000157 *x*_1_ − 0.0000008 *x*_1_^2^ − 0.020901 *x*_2_ + 0.000454 *x*_2_^2^ + 0.000016 *x*_1_*x*_2_

Similarly, the quadratic models were significant for the heterotrophic conditions (*p* < 0.05), showing coefficients of determination (R^2^) of 0.541 and 0.566 for biomass and lipid production, respectively (Table S2; Figure S2).

*y*_(biomass)_ = 1.391449 + 0.117238 *x*_1_ − 0.003264 *x*_1_^2^ − 0.133289 *x*_2_ + 0.004450 *x*_2_^2^ − 0.001026 *x*_1_*x*_2_

*y*_(lipid)_ = −0.053182 + 0.040369 *x*_1_ − 0.001095 *x*_1_^2^ − 0.005486 *x*_2_ + 0.000474 *x*_2_^2^ − 0.000409 *x*_1_*x*_2_

**Figure 2:**
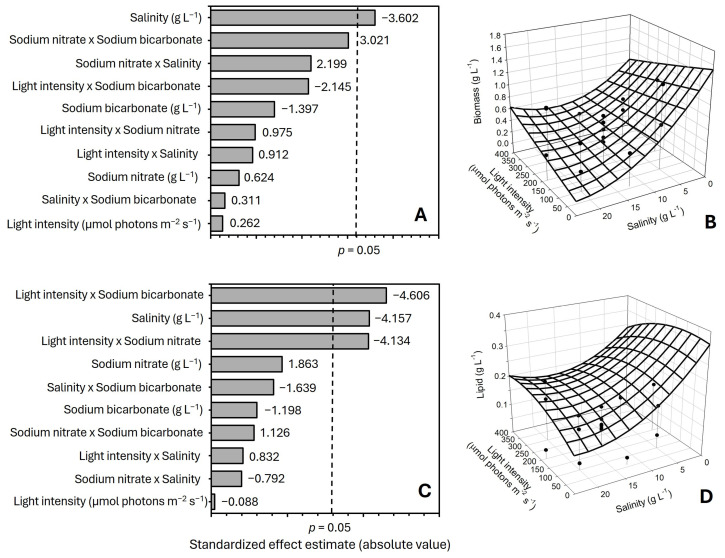
Pareto charts of standardized effects and fitted response surface plots obtained from the central composite rotatable design (CCRD) applied to autotrophic cultivation conditions. The Pareto charts show the significant linear, and interaction effects of light intensity, salinity (NaCl), nitrogen concentration, and sodium bicarbonate on biomass (**A**) and lipid production (**C**). The response surface plots illustrate the combined influence of the most significant variables on biomass (g L^−1^; (**B**)) and lipid yields (g L^−1^; (**D**)), and the black circles represent the measured values. The vertical reference line in the Pareto charts represents the statistical significance threshold at *p* = 0.05.

Under illuminated autotrophic conditions (Figure 2), biomass formation was primarily dictated by salinity, which showed a strong inhibitory linear effect accompanied by a significant positive quadratic contribution (Figure 2A,B) [31]. This behavior suggests that *N. oleoabundans* tolerate salinity within a narrow curvature-dependent range [32]. The strong significance of the salinity-nitrogen interaction demonstrates that nitrogen availability can partially offset salt stress possibly by supporting the synthesis of compatible solutes and stress-response proteins thus enabling growth under intermediate salinity. In contrast, light incidence and nitrogen alone exerted relatively weak individual effects within the tested domain, indicating that neither variable was growth-limiting when salinity dominated the physiological response.

Unlike biomass, lipid accumulation was not primarily governed by salinity but instead by the interaction between light incidence and nitrogen, along with significant curvature effects in both nitrogen and sodium bicarbonate (Figure 2C,D) [21]. These results indicate that lipid accumulation in *N. oleoabundans* is less dependent on osmotic stress and more strongly driven by nutrient–light interactions. High irradiance coupled with moderate-to-low nitrogen likely imposes a redirection of excess photosynthetic reducing power toward storage lipids, a classical stress-induced mechanism. The strong quadratic effects observed for bicarbonate and nitrogen reinforce the presence of metabolic “transition zones”, where cells switch from growth-oriented to carbon-storage phenotypes [12].

**Figure 3:**
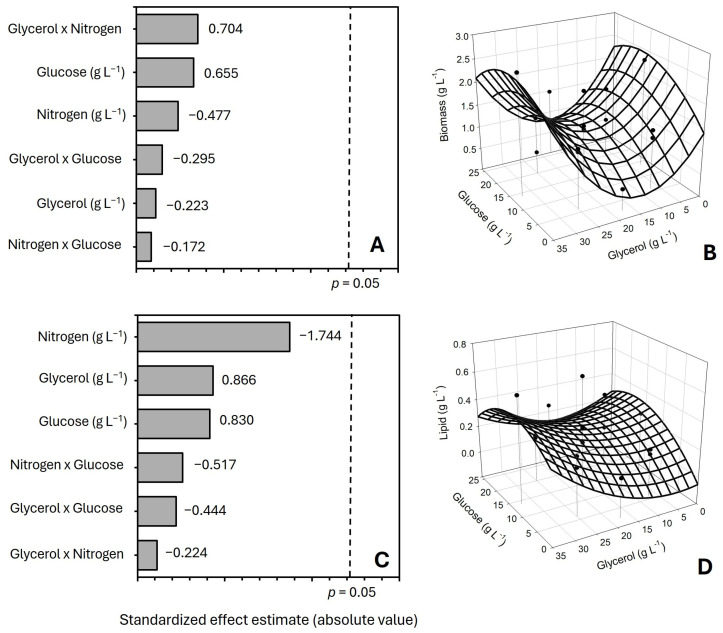
Pareto charts of standardized effects and fitted response surface plots obtained from the central composite rotatable design (CCRD) applied to heterotrophic cultivation conditions. The Pareto charts present the significant linear, and interaction effects of glucose, glycerol, and nitrogen concentration on biomass (**A**) and lipid production (**C**). The response surface plots illustrate the combined effects of the most influential variables on biomass (g L^−1^; (**B**)) and lipid yields (g L^−1^; (**D**)), and the black circles represent the measured values. The vertical reference line in the Pareto charts represents the statistical significance threshold at *p* = 0.05.

Under heterotrophic conditions (Figure 3), carbon availability emerged as the primary driver of biomass formation (Figure 3A,B) [33]. Nitrogen, although essential, exerted only modest influence on growth within the evaluated range. Biomass was mostly achieved under high carbon conditions, aligning with the metabolism of oleaginous microalgae, which is known to prioritize rapid growth when assimilable carbon is abundant.

In contrast, lipid accumulation under heterotrophy followed the opposite trend: Nitrogen became the most influential factor, exhibiting a strong negative linear effect on lipid accumulation (Figure 3C,D). Glycerol also contributed positively to lipid accumulation, possibly serving not only as a carbon substrate but also as an indirect metabolic enhancer for TAG assembly [34]. The response surfaces revealed that the highest lipid percentages in algal biomass occurred under low nitrogen and high carbon availability, consistent with established regulatory mechanisms in oleaginous microorganisms.

Together, the two CCRDs delineate the multidimensional interplay between salinity, light, nitrogen, bicarbonate, and carbon-source composition in shaping growth and lipid accumulation. Conditions that maximize biomass did not coincide with those that maximized lipid accumulation. Thus, by identifying nonlinearities, interaction-driven behaviors, and curvature-dependent metabolic transitions, the models provide a precise roadmap for optimization. The clear divergence between growth- and lipid-driven conditions also emphasizes that bioprocess strategies for *N. oleoabundans* must explicitly account for the metabolic regime autotrophic/mixotrophic vs. heterotrophic when defining targets for industrial lipid production. These insights form a robust foundation for the next phase of model refinement and process integration.

The literature reports somewhat heterogenous results, as microalgae species and cultivation conditions vary widely across studies. For example, in line with our results, Das et al. [35] showed that the heterotrophic growth of the newly isolated cyanobacterium *Leptolyngbya subtilis* accumulated substantially more lipids compared to its autotrophic growth using glycerol as carbon source. In contrast, the freshwater green microalga *Chlorella vulgaris*, and the marine diatom *Cyclotella cryptica* produced more lipids under photoautotrophic growth [36,37]. Whereas the former presented a higher diversity of lipids under autotrophic conditions, the latter had higher EPA productivity under heterotrophic conditions. To the best of our knowledge, this is the first time the autotrophic/heterotrophic conditions are investigated to accumulate lipids in *N. oleoabundans*.

### 3.2. Microalgae Cultivation and Nile Red Staining

**Figure 4:**
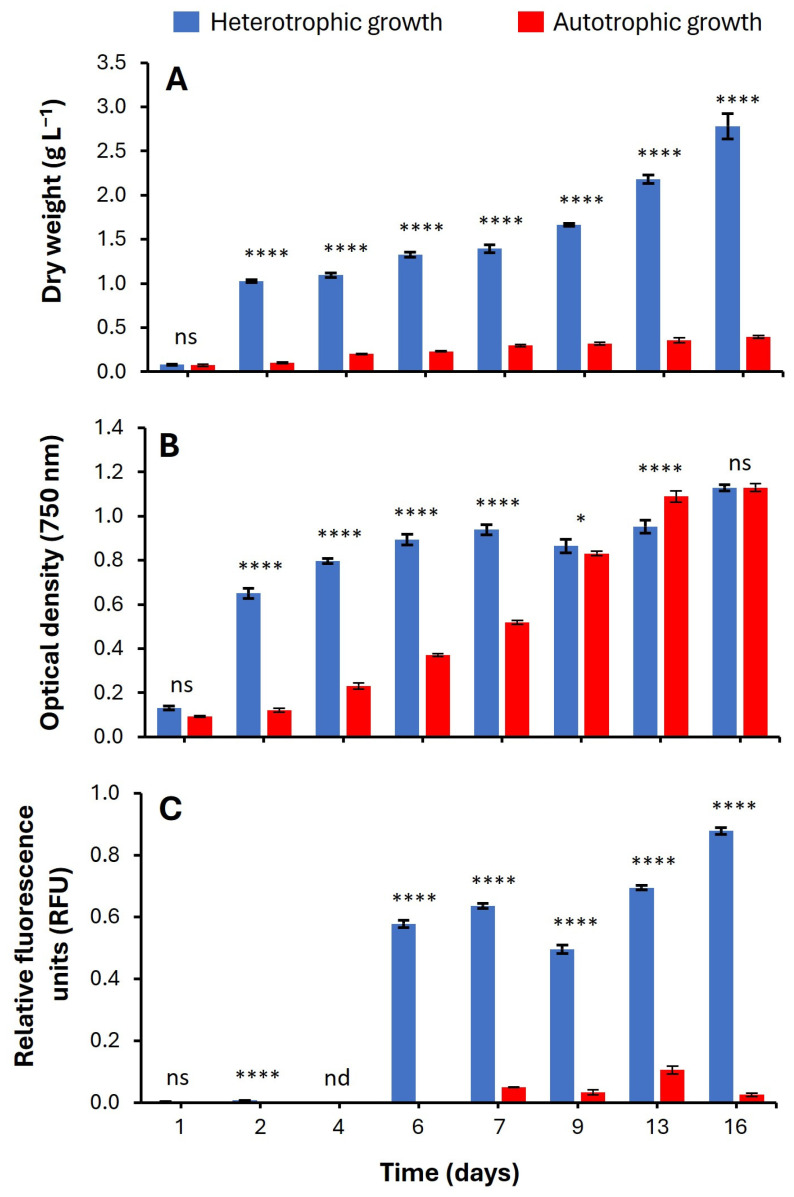
Time-course profiles of biomass dry weight (**A**), optical density (**B**), and relative fluorescence units (**C**) as an indicator of intracellular neutral lipid accumulation under autotrophic (red bars) and heterotrophic (blue bars) cultivation. Heterotrophic cultivation resulted in faster biomass accumulation, and a marked increase in fluorescence intensity over time, indicating enhanced lipid accumulation. Autotrophic conditions showed comparatively lower growth but distinct lipid accumulation trends over time. The reported data are the means of three to five independent replicates (3 ≤ *n* ≤ 5) and error bars show standard deviation. Asterisks show statistical significance where * = *p* < 0.05; and **** = *p* < 0.0001. Abbreviations: ns = not significant; nd = not determined.

A comparative analysis of the cultures grown under the conditions that yielded the highest biomass was performed by examining their growth kinetics through optical density and dry weight measurements (Figure 4A,B). The heterotrophic culture exhibited a significantly faster growth, reaching the stationary phase as early as day 2. In contrast, the autotrophic culture showed a substantially slower growth and had not yet reached the stationary phase even at the end of the 16-day evaluation period. This divergence is further reflected in the dry weight data, which reveals a pronounced and statistically significant difference between the two metabolic modes (*p* < 0.0001). Heterotrophic cultivation achieved a maximum dry biomass concentration of 2.78 ± 0.14 g L^−1^, whereas autotrophic cultivation reached 0.39 ± 0.01 g L^−1^.

**Figure 5:**
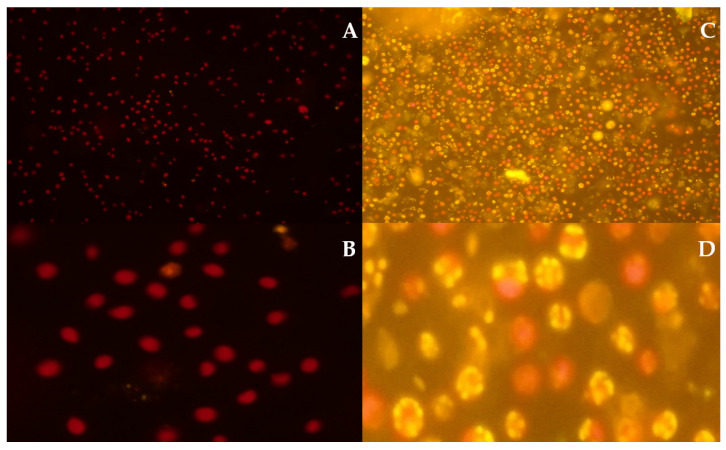
Fluorescence microscopy was used to evaluate the intracellular lipid composition of *Neochloris oleoabundans* cultivated under autotrophic and heterotrophic conditions for 16 days. In the autotrophic culture (**A**,**B**), cells exhibited predominantly red fluorescence, corresponding to polar lipid staining, whereas the heterotrophic culture (**C**,**D**) displayed a substantially higher abundance of yellow–orange fluorescence, indicative of neutral lipid accumulation. These contrasting fluorescence patterns reflect the distinct metabolic responses of *N. oleoabundans* to nutrient availability and carbon source supply during cultivation. 60× magnification.

Fluorescence microscopy demonstrated that *N. oleoabundans* exhibits markedly different lipid profiles depending on the cultivation strategy (Figure 4C and Figure 5). The abundance and intensity of these signals reflect the metabolic reorientation. In the autotrophic culture cells displayed predominantly red fluorescence, corresponding to the staining of polar lipids typically associated with photosynthetic and structural membranes (Figure 5A,B). The scarcity of yellow–orange emission, indicative of neutral, non-polar lipids such as TAGs, suggests that under these conditions the cells accumulated only minimal amounts of storage lipids. This result displays the typical metabolic behavior of microalgae grown under sufficient light and balanced nutrients, which preferentially channel carbon toward membrane formation and protein synthesis rather than lipid storage [38,39].

In contrast, the heterotrophic culture exhibited a much higher density of cells and a remarkably strong yellow-orange fluorescence, characteristic of elevated accumulation of neutral lipids, within cytosolic lipid bodies (Figure 5C,D). The abundance and intensity of these signals reflect metabolic reprogramming toward lipid storage under heterotrophic conditions. Heterotrophic metabolism enhances the flux of carbon toward acetyl-CoA and subsequently fatty acid synthesis, particularly once nitrogen becomes limiting. This shift leads to enhanced TAG assembly and the formation of prominent lipid bodies, a hallmark observed in several oleaginous microalgae under similar conditions [40,41,42].

Overall, these observations demonstrate that heterotrophic cultivation significantly enhances neutral lipid accumulation, whereas autotrophic growth favors structural lipid synthesis in *N. oleoabundans*. This distinction is particularly relevant for applications targeting non-polar lipids of industrial relevance such as precursors for emulsifiers since heterotrophic conditions provide a more efficient route for maximizing TAG accumulation.

### 3.3. Lipid Extraction and Analysis

Four extraction methods were compared to assess the lipid profile of *N. oleoabundans*: (i) hexane extraction, which preferentially solubilizes non-polar lipids (neutral lipids, e.g., TAGs, FFAs) due to its low polarity; (ii) methanol:hexane (1:1 *v*/*v*), a biphasic-favoring mixture that increases the solubilization range to include some amphipathic lipids while still efficiently extracting neutral fractions; (iii) methanol:hexane (5:1 *v*/*v*), a more polar mixture that improves recovery of polar and strongly associated lipid species (e.g., glycolipids, phospholipids) compared with pure hexane; and (iv) the Bligh and Dyer procedure (chloroform:methanol:water system), a classical monophasic-biphasic method designed to quantitatively extract total lipids, including polar membrane lipids and neutral storage lipids, by virtue of exhaustive solvent partitioning. The Folch and Bligh–Dyer protocols remain standard references for comprehensive lipid extraction and partitioning of polar vs. non-polar lipid classes.

**Figure 6:**
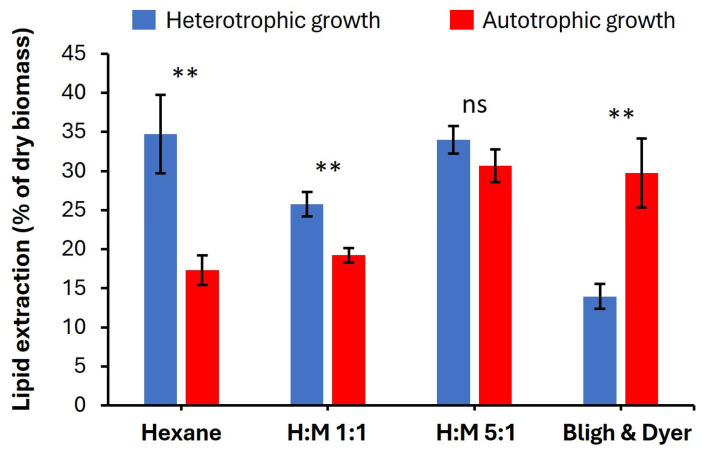
Lipid extraction (in % of dry biomass) obtained using different extraction methods after autotrophic (red bars) and heterotrophic (blue bars) growth. Variations in extraction efficiency reflect the influence of solvent composition and polarity on lipid recovery, with differences observed between samples derived from autotrophic and heterotrophic cultivations. The reported data are the means of three independent replicates (*n* = 3) and error bars show standard deviation. Asterisks show statistical significance where ** = *p* < 0.01. Abbreviation: ns = not significant.

Under heterotrophic growth, lipid extraction varied substantially among solvents (Figure 6). Pure hexane yielded the highest extraction (35%), with highly significant differences compared to other solvents. This result confirms that heterotrophically cultivated *N. oleoabundans* predominantly accumulates neutral lipids, particularly TAGs, which are efficiently solubilized by non-polar solvents. The hexane:methanol mixture (5:1) produced a comparable extraction (34%), indicating that low-polarity systems are sufficient to recover TAG-rich fraction.

In contrast, the hexane:methanol 1:1 mixture exhibited reduced extraction (26%), confirming that increased solvent polarity compromises recovery of hydrophobic lipid classes. The Bligh and Dyer method, optimized for wet biomass and polar lipids, showed the lowest recovery (14%), reinforcing that classical chloroform-methanol extraction is suboptimal for dried, TAG-rich heterotrophic cells.

Autotrophically cultivated biomass displayed a markedly different extraction pattern. Pure hexane extracted 17% lipids from the dry biomass, confirming the lower abundance of neutral lipids compared to heterotrophic cells. Extraction improved with the H:M 1:1 mixture (25%), reflecting enhanced solubilization of membrane-associated polar lipids. The H:M 5:1 mixture (30%) significantly improved extraction relative to hexane alone, although the difference from polar extraction was not statistically significant. The Bligh and Dyer method achieved the highest extraction (30%), outperforming all other solvents, consistent with the well-established affinity of chloroform:methanol mixtures for polar lipids, including phospholipids, glycolipids, and chloroplast-derived galactolipids.

These solvent–biomass interactions are consistent with previous findings showing that trophic conditions fundamentally reshape the lipidome of *N. oleoabundans* [43,44]. Heterotrophic metabolism promotes TAG accumulation due to carbon excess and oxygen limitation, whereas autotrophic cultures maintain high polar-lipid content for thylakoid membrane maintenance.

Several lipid extraction methods and solvents have been investigated in numerous microalgae species and strains. Zarrinmehr et al. [45] demonstrated that the polarity of solvents and lipid extraction conditions dramatically affected the amount and composition of lipids extracted from the green microalgae *Scenedesmus quadricauda*. In contrast, Meroiço et al. [46] found no differences in lipid extraction yields when investigating polar:apolar solvent ratios in the green microalgae *Tetradesmus obliquus*. Promising results were reported on more recent methods, such as ultrasound assisted extraction and deep eutectic solvents in other microalgae [47,48]. However, the great variability in procedures, reagents, and microalgae species/strains used makes generalizations extremely difficult.

**Figure 7:**
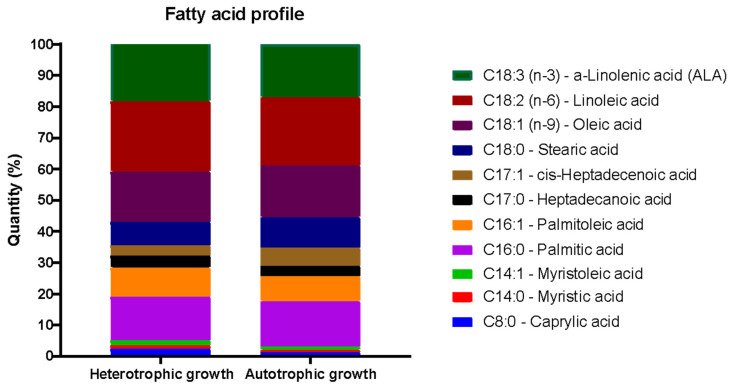
Lipid profile of the microalga *Neochloris oleoabundans* showing the relative abundance of the main lipid classes identified in the samples. The bar charts represent the proportional distribution of lipid fractions, while the legend indicates the corresponding lipid categories. Data are presented as relative percentage composition of total lipids. The data represents a descriptive single measurement of pooled microalgal oil sample (*n* = 1).

PUFAs, monounsaturated fatty acids (MUFAs), and saturated fatty acids (SFAs) were differentially distributed across the two growth modes (Figure 7), reflecting metabolic adjustments associated with carbon availability, energy balance, and cellular redox state. PUFAs dominated the profile in both conditions, but with distinct patterns. Under heterotrophic cultivation, linoleic acid (C18:2 *n*-6) and oleic acid (C18:1 *n*-9) were markedly elevated relative to autotrophy, indicating enhanced flux through the Δ9- and Δ12-desaturation pathways [49]. This is consistent with reports showing that reduced light and increased organic carbon supply promote accumulation of storage lipids enriched in C18-based MUFAs and *n*-6 PUFAs [50]. α-Linolenic acid (C18:3 *n*-3), although present in both cultures, was noticeably more abundant under autotrophic conditions, supporting the well-established light-dependent regulation of *n*-3 PUFA desaturases [51].

Autotrophic cultures also displayed comparatively higher proportions of saturated and medium-chain fatty acids such as palmitic acid (C16:0), myristic acid (C14:0), and caprylic acid (C8:0). This pattern aligns with the role of de novo fatty acid synthesis in chloroplasts, where SFA production is favored under high light and photosynthetic activity [52]. The elevated levels of C18:0 (stearic acid) in the autotrophic samples further suggest limited elongation–desaturation turnover relative to heterotrophic cells [53].

Heterotrophic growth, in contrast, favored MUFA production, with consistently higher quantities of palmitoleic acid (C16:1) and oleic acid (C18:1). Such enrichment is characteristic of lipid bodies formed during growth on organic carbon sources, where acetyl-CoA availability and NADPH supply support rapid synthesis of storage TAGs enriched in MUFAs [54]. The increased C18:2 (*n*-6) fraction further indicates active Δ12-desaturation, a hallmark of carbon-rich heterotrophic conditions. However, these mechanistic reasons were not directly investigated in this study.

Interestingly, odd-chain fatty acids (C17:0 and C17:1) were detected at low but measurable levels in both trophic modes, suggesting propionyl-CoA incorporation into fatty acid biosynthesis, a phenomenon previously reported for several microalgal species. Collectively, the results show that autotrophic cultures favor *n*-3 PUFA and SFA enrichment, whereas heterotrophic cultures accumulate MUFAs and *n*-6 PUFAs, a divergence consistent with central metabolic rewiring between photosynthetic and carbon-fed growth [55]. These findings are important for tailoring cultivation strategies depending on whether the target is nutritional lipids (*n*-3 PUFA-rich profiles) or industrial lipids (MUFA-rich profiles well-suited for biodiesel and oleochemical applications).

### 3.4. MAG and DAG Formation

**Figure 8:**
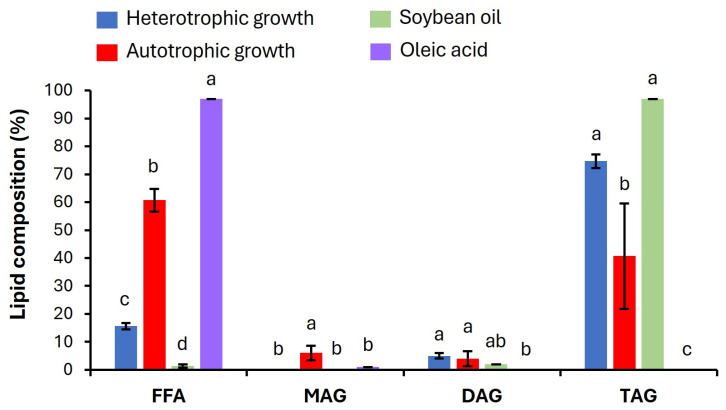
Compositional profile of the oils used in this study. Comparison among microalgal oil obtained under autotrophic cultivation (red bars), microalgal oil under heterotrophic cultivation (blue bars), soybean oil (green bars), and free fatty acids (purple bars). Data is presented as the relative distribution of the main lipid components. The reported data represent the means of three independent replicates (*n* = 3) and error bars show standard deviation. Different letters above bars represent statistically significant differences within the same lipid class (*p* < 0.05).

Autotrophically grown microalgae exhibited a lipid profile dominated by FFAs (60%), with only minor fractions of MAGs (6%), DAGs (4%), and TAGs (41%) (Figure 8). The high FFA content is consistent with previous reports showing that photoautotrophic cultures often accumulate enhanced experience of lipolysis under nutrient stress or photoinhibition, leading to partial hydrolysis of storage lipids [43,56]. The relatively modest TAG fraction suggests limited channeling of carbon toward storage lipids under photosynthetic growth; a trend commonly observed in microalgae when light and nutrient conditions fluctuate.

In contrast, heterotrophically cultivated microalgae exhibited a TAG-rich lipid profile, reflecting enhanced carbon flux toward storage lipid biosynthesis (75%), with markedly reduced FFA levels (15%) (Figure 8). This TAG-rich phenotype reflects the activation of de novo lipogenesis mechanisms, widely documented in oleaginous microalgae under organic carbon feeding [57].

Soybean oil, used as a conventional reference lipid, shows the expected profile consisting almost exclusively of TAGs (98%), with negligible MAG, DAG, or FFA fractions consistent with its refined and highly esterified nature. This sample serves as a benchmark for evaluating the structural lipid quality of microalgae oils.

Finally, oleic acid presents a profile composed almost entirely of FFA (100%), confirming the purity of the substrate and serving as a baseline for FFA-rich systems. Collectively, these results demonstrate that heterotrophic cultivation yields microalgae oils whose lipid-class distribution closely approaches that of commercial plant oils, whereas autotrophic oils present a more hydrolyzed and heterogeneous composition. This distinction has important implications for downstream biocatalysis, emulsifier synthesis, and lipid-based biorefinery strategies, since acylglycerol composition strongly determines the efficiency of processes such as glycerolysis, interesterification, and lipase-mediated structuring.

**Figure 9:**
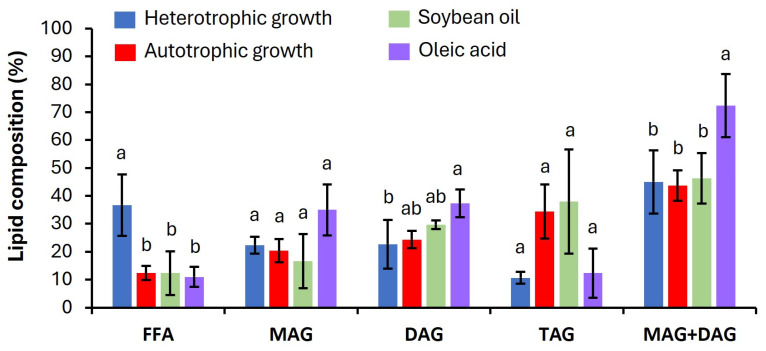
Compositional profile of the products after enzymatic catalysis using lipase. Formation of monoacylglycerols (MAG) and diacylglycerols (DAG) from autotrophic microalgal oil (red bars), heterotrophic microalgal oil (blue bars), soybean oil (green bars), and free fatty acids (purple bars). Results highlight differences in conversion efficiency among the feedstocks. The reported data represent the means of three independent replicates (*n* = 3) and error bars show standard deviation. Different letters above bars represent statistically significant differences within the same lipid class (*p* < 0.05).

Glycerolysis reactions catalyzed by Novozym 435 resulted in distinct product distributions depending on substrate composition. Across all reactions, the formation of MAGs, DAGs, and residual TAGs follows the expected equilibrium-driven behavior of lipase-catalyzed glycerolysis. However, clear differences emerge among the four feedstocks (Figure 9). Autotrophic microalgae oil produced a balanced distribution of MAG + DAG (43%), with an intermediate amount of remaining FFA (12%) and TAG (34%). The presence of FFA reflects the high initial FFA content of the crude autotrophic oil, which underwent no neutralization or refining. Elevated FFAs are known to interfere with the adsorption of glycerol on the lipase active site and may partially inhibit the catalyst or shift the reaction toward esterification rather than glycerolysis [58]. Despite this, the results suggest that Novozym 435 maintained good catalytic performance even in the presence of impurities and polar lipids typical of photosynthetic microalgal extracts.

The heterotrophic microalgae oil characterized by a TAG-rich and low-FFA profile prior to reaction displayed a slightly different conversion pattern, with TAG conversion in MAG + DAG (45%) and hydrolysis to FFA (37%). This behavior aligns with the greater structural similarity between heterotrophic microalgae oil and refined plant oils, allowing more favorable substrate-enzyme interactions. However, the presence of pigments, phospholipids, and minor cellular residues expected in non-refined microalgae oils likely reduced MAG yields relative to refined soybean oil. These impurities can adsorb onto the hydrophobic surface of the immobilized lipase, thereby reducing catalytic efficiency [59,60,61].

Soybean oil, which served as a refined, low-impurity standard, contrary to what was expected, marked low yields of MAG (16%) DAG (30%), and FFA (12%) formation and substantial TAG depletion. In contrast, oleic acid composed almost entirely of FFAs yielded the highest relative levels of MAG + DAG among all substrates arising predominantly from esterification rather than glycerolysis. Because FFAs must first undergo lipase-catalyzed esterification with glycerol to form MAGs (followed by further acylation to form DAGs), the reaction pathway differs fundamentally from TAG glycerolysis. The oleic-acid system generated a high MAG + DAG pool (72%), but with lower TAG formation, reflecting the absence of initial triacylglycerols. This conversion pattern is consistent with literature reporting that lipid systems rich in FFAs favor esterification routes and often lead to high MAG yields when glycerol availability is high [62].

Taken together, the data demonstrates that substrate purity, acylglycerol class distribution, and cultivation-specific lipid architecture may influence the efficiency of Novozym 435 in glycerolysis. Non-refined microalgae oils, especially those from autotrophic and heterotrophic cultivation, contain FFAs, pigments, and polar lipids that modulate catalytic behavior; while oleic acid as substrate facilitated lipase–substrate interactions to MAG + DAG formation. These findings demonstrate that, in addition to the need for optimization of the parameters governing MAG and DAG formation across the different substrates evaluated, refining the microalgae extracted oils can substantially improve catalytic performance by reducing the presence of interfering compounds.

## 4. Conclusions

This work demonstrates that microalgal cultivation strategies can be engineered to produce lipid feedstocks with tailored biochemical properties for enzymatic upgrading. It shifts perspective from maximizing lipid accumulation to engineering lipid functionality, highlighting that lipid class distribution and composition critically determine catalytic performance.

The results revealed a clear relationship between trophic cultivation mode and lipid reactivity. Heterotrophic cultivation resulted in TAG-rich oils, closely resembling conventional vegetable oils and enabling higher conversion efficiencies into MAGs and DAGs. In contrast, autotrophic oils were characterized by elevated levels of FFAs and polar lipids, which negatively affected enzymatic performance and favored alternative reaction pathways.

Importantly, microalgal cultivation is repositioned here as a tool for designing reactive lipid feedstocks rather than merely producing biomass. The identification of a metabolic trade-off between biomass productivity and lipid reactivity further underscores that optimal process conditions must be defined according to downstream application requirements rather than lipid yield alone. Moreover, the results demonstrate that substrate composition dictates not only conversion yield but also the dominant catalytic pathway, emphasizing the need to consider lipid architecture when designing enzymatic processes involving complex biological feedstocks.

Overall, the integration of cultivation strategy with enzymatic conversion represents a critical step toward the development of truly integrated microalgal biorefineries. By establishing a direct link between upstream metabolic control and downstream biocatalytic efficiency, this study provides a conceptual and practical framework for advancing microalgae-based platforms toward high-value applications in food, nutraceutical, and oleochemical industries.

## Figures and Tables

**Table 1:** Experimental design employed in this study: a Plackett–Burman design for variable screening (glucose, glycerol, sodium acetate, sodium bicarbonate, dipotassium phosphate, salinity, and light intensity).

Level	Glucose (g L^−1^)	Glycerol (gL^−1^)	Sodium Acetate (g L^−1^)	Sodium Bicarbonate (g L^−1^)	Dipotassium Phosphate (g L^−1^)	Salinity (g_NaCl_ L^−1^)	Light Intensity (μmol Photons m^−2^ s^−1^)
−1	0	0	0	0	0.75	0.25	50
0	1.5	2	1	1.5	1.5	1.38	75
1	3	4	2	3	2.75	2.5	100

**Table 2:** Central composite rotational design (CCRD) applied to autotrophic cultivation, evaluating the effects of light intensity, salinity, nitrogen, and sodium bicarbonate. Both coded and actual factor levels are presented for each experimental condition.

Level	Light Intensity (μmol Photons m^−2^ s^−1^)	Salinity (g_NaCl_ L^−1^)	Nitrogen (g L^−1^)	Sodium Bicarbonate (g L^−1^)
−1.41	59	1.9	0.05	0.29
−1	100	5.0	0.15	0.50
0	200	12.5	0.30	1.00
1	300	20.0	0.50	1.50
1.41	341	23.1	0.55	1.71

**Table 3:** Central composite rotational design (CCRD) applied to heterotrophic cultivation, considering glucose, glycerol, and nitrogen as independent variables. Both coded and actual factor levels are presented for each experimental condition.

Level	Glucose (g L^−1^)	Glycerol (g L^−1^)	Nitrogen (g L^−1^)
−1.41	2.4	2.2	0.08
−1	6.0	7.0	0.25
0	14.0	17.5	0.50
1	20.0	25.0	0.75
1.41	25.8	32.6	0.92

## Data Availability

The original contributions presented in this study are included in the article/Supplementary Materials. Further inquiries can be directed to the corresponding author.

## Supplementary Materials

The following supporting information can be downloaded at: https://www.mdpi.com/article/10.3390/foods15132333/s1, Table S1: ANOVA tables from the central composite rotatable design (CCRD) applied to autotrophic cultivation conditions. Table S2: ANOVA tables from the central composite rotatable design (CCRD) applied to heterotrophic cultivation conditions. Figure S1: CCRD observed x predicted values for biomass (a) and lipid (b) production under autotrophic conditions. Figure S2: CCRD observed x predicted values for biomass (a) and lipid (b) production under heterotrophic conditions.

## References

[1] The United Nations (2024). World Population Prospects 2024: Summary of Results.

[2] Savilaakso S., Laumonier Y., Guariguata M.R., Nasi R. (2013). Does Production of Oil Palm, Soybean, or Jatropha Change Biodiversity and Ecosystem Functions in Tropical Forests. Environ. Evid..

[3] Meijaard E., Brooks T.M., Carlson K.M., Slade E.M., Garcia-Ulloa J., Gaveau D.L.A., Lee J.S.H., Santika T., Juffe-Bignoli D., Struebig M.J. (2020). The Environmental Impacts of Palm Oil in Context. Nat. Plants.

[4] Camacho F.G., Grima E.M., Mirón A.S., Pascual V.G., Chisti Y. (2001). Carboxymethyl Cellulose Protects Algal Cells against Hydrodynamic Stress. Enzym. Microb. Technol..

[5] Khoo K.S., Chew K.W., Yew G.Y., Leong W.H., Chai Y.H., Show P.L., Chen W.H. (2020). Recent Advances in Downstream Processing of Microalgae Lipid Recovery for Biofuel Production. Bioresour. Technol..

[6] Xu Q., Tang Q., Xu Y., Wu J., Mao X., Li F., Wang S., Wang Y. (2025). Biotechnology in Future Food Lipids: Opportunities and Challenges. Annu. Rev. Food Sci. Technol..

[7] Ferreira G.F., Pessoa J.G.B., Ríos Pinto L.F., Maciel Filho R., Fregolente L.V. (2021). Mono- and Diglyceride Production from Microalgae: Challenges and Prospects of High-Value Emulsifiers. Trends Food Sci. Technol..

[8] Xue Z., Yu Y., Yu W., Gao X., Zhang Y., Kou X. (2020). Development Prospect and Preparation Technology of Edible Oil from Microalgae. Front. Mar. Sci..

[9] Conde T.A., Neves B.F., Couto D., Melo T., Neves B., Costa M., Silva J., Domingues P., Domingues M.R. (2021). Microalgae as Sustainable Bio-Factories of Healthy Lipids: Evaluating Fatty Acid Content and Antioxidant Activity. Mar. Drugs.

[10] Freitas L., Bueno T., Perez V.H., De Castro H.F. (2008). Monoglicerídeos: Produção Por via Enzimática e Algumas Aplicações. Quim. Nova.

[11] Lee W.J., Zhang Z., Lai O.M., Tan C.P., Wang Y. (2020). Diacylglycerol in Food Industry: Synthesis Methods, Functionalities, Health Benefits, Potential Risks and Drawbacks. Trends Food Sci. Technol..

[12] Safi C., Pollio A., Olivieri G. (2021). *Neochloris oleoabundans* from Nature to Industry: A Comprehensive Review. Rev. Environ. Sci. Biotechnol..

[13] Rashidi B., Trindade L.M. (2018). Detailed Biochemical and Morphologic Characteristics of the Green Microalga *Neochloris oleoabundans* Cell Wall. Algal Res..

[14] Foley P.M., Beach E.S., Zimmerman J.B. (2011). Algae as a Source of Renewable Chemicals: Opportunities and Challenges. Green Chem..

[15] Popovich C.A., Damiani C., Constenla D., Martínez A.M., Freije H., Giovanardi M., Pancaldi S., Leonardi P.I. (2012). *Neochloris oleoabundans* Grown in Enriched Natural Seawater for Biodiesel Feedstock: Evaluation of Its Growth and Biochemical Composition. Bioresour. Technol..

[16] Banerjee S., Singh H., Das D., Atta A. (2019). Process Optimization for Enhanced Biodiesel Production by Neochloris oleoabundans UTEX 1185 with Concomitant CO2 Sequestration. Ind. Eng. Chem. Res..

[17] Del Castillo-Santaella T., Maldonado-Valderrama J., Cabrerizo-Vílchez M.Á., Rivadeneira-Ruiz C., Rondón-Rodriguez D., Gálvez-Ruiz M.J. (2015). Natural Inhibitors of Lipase: Examining Lipolysis in a Single Droplet. J. Agric. Food Chem..

[18] Dąbrowski G., Czaplicki S., Szustak M., Korkus E., Gendaszewska-Darmach E., Konopka I. (2024). The Impact of Selected Xanthophylls on Oil Hydrolysis by Pancreatic Lipase: In Silico and in Vitro Studies. Sci. Rep..

[19] Andersen R.A. (2004). Algal Culturing Techniques.

[20] Baldisserotto C., Sabia A., Ferroni L., Pancaldi S. (2019). Biological Aspects and Biotechnological Potential of Marine Diatoms in Relation to Different Light Regimens. World J. Microbiol. Biotechnol..

[21] Li J., Li C., Lan C.Q., Liao D. (2018). Effects of Sodium Bicarbonate on Cell Growth, Lipid Accumulation, and Morphology of Chlorella Vulgaris. Microb. Cell Fact..

[22] Teh K.Y., Loh S.H., Aziz A., Takahashi K., Effendy A.W.M., Cha T.S. (2021). Lipid Accumulation Patterns and Role of Different Fatty Acid Types towards Mitigating Salinity Fluctuations in Chlorella Vulgaris. Sci. Rep..

[23] Tomabene T.G., Holzer G., Lien S., Burris N. (1983). Lipid Composition of the Nitrogen Starved Green Alga Neochloris oleoabundans. Enzym. Microb. Technol..

[24] Alemán-Nava G.S., Cuellar-Bermudez S.P., Cuaresma M., Bosma R., Muylaert K., Ritmann B.E., Parra R. (2016). How to Use Nile Red, a Selective Fluorescent Stain for Microalgal Neutral Lipids. J. Microbiol. Methods.

[25] Bligh E.G., Dyer W.J. (1959). A rapid method of total lipid extraction and purification. Can. J. Biochem. Physiol..

[26] Cavalcanti-Oliveira E.D., Silva P.R., Rosa T.S., Moura N.M.L., Santos B.C.P., Carvalho D.B., Sousa J.S., Carvalhinho M.T.J.E., Castro A.M., Freire D.M.G. (2015). Methods to prevent acidification of Macaúba (*Acrocomia aculeata*) fruit pulp oil: A promising oil for producing biodiesel. Ind. Crops Prod..

[27] (2024). Fat and Oil Derivatives—Fatty Acid Methyl Esters (FAME)—Determination of Free and Total Glycerol and Mono-, Di-, Triglyceride Contents.

[28] Kim T.H., Lee Y., Han S.H., Hwang S.J. (2013). The Effects of Wavelength and Wavelength Mixing Ratios on Microalgae Growth and Nitrogen, Phosphorus Removal Using Scenedesmus Sp. for Wastewater Treatment. Bioresour. Technol..

[29] Silva H.R., Prete C.E.C., Zambrano F., de Mello V.H., Tischer C.A., Andrade D.S. (2016). Combining Glucose and Sodium Acetate Improves the Growth of *Neochloris oleoabundans* under Mixotrophic Conditions. AMB Express.

[30] Baldisserotto C., Ferroni L., Giovanardi M., Boccaletti L., Pantaleoni L., Pancaldi S. (2012). Salinity Promotes Growth of Freshwater Neochloris oleoabundans UTEX 1185 (Sphaeropleales, Chlorophyta): Morphophysiological Aspects. Phycologia.

[31] de Jaeger L., Carreres B.M., Springer J., Schaap P.J., Eggink G., Santos V.A.P.M., Dos, Wijffels R.H., Martens D.E. (2018). *Neochloris oleoabundans* Is Worth Its Salt: Transcriptomic Analysis under Salt and Nitrogen Stress. PLoS ONE.

[32] Santos A.M., Janssen M., Lamers P.P., Evers W.A.C., Wijffels R.H. (2012). Growth of Oil Accumulating Microalga *Neochloris oleoabundans* under Alkaline-Saline Conditions. Bioresour. Technol..

[33] Morales-Sánchez D., Tinoco-Valencia R., Kyndt J., Martinez A. (2013). Heterotrophic Growth of *Neochloris oleoabundans* Using Glucose as a Carbon Source. Biotechnol. Biofuels.

[34] Chen Y.H., Walker T.H. (2011). Biomass and Lipid Production of Heterotrophic Microalgae Chlorella Protothecoides by Using Biodiesel-Derived Crude Glycerol. Biotechnol. Lett..

[35] Das S., Nath K., Chowdhury R. (2021). Comparative studies on biomass productivity and lipid content of a novel blue-green algae during autotrophic and heterotrophic growth. Environ. Sci. Pollut. Res. Int..

[36] Couto D., Melo T., Conde T.A., Costa M., Silva J., Domingues M.R.M., Domingues P. (2021). Chemoplasticity of the polar lipid profile of the microalgae *Chlorella vulgaris* grown under heterotrophic and autotrophic conditions. Algal Res..

[37] Cupo A., Landi S., Morra A., Nuzzo G., Gallo C., Manzo E., Fontana A., d’Ippolito G. (2021). Autotrophic vs. heterotrophic cultivation of the marine diatom Cyclotella cryptica for EPA production. Mar. Drugs.

[38] Greenspan P., Mayer E.P., Fowler S.D. (1985). Nile Red” A Selective Fluorescent Stain for Intracellular Lipid Droplets. J. Cell Biol..

[39] Pick U., Rachutin-Zalogin T. (2012). Kinetic Anomalies in the Interactions of Nile Red with Microalgae. J. Microbiol. Methods.

[40] Cavonius L.R., Carlsson N.G., Undeland I. (2014). Quantification of Total Fatty Acids in Microalgae: Comparison of Extraction and Transesterification Methods. Anal. Bioanal. Chem..

[41] Tan K.W.M., Lee Y.K. (2016). The Dilemma for Lipid Productivity in Green Microalgae: Importance of Substrate Provision in Improving Oil Yield without Sacrificing Growth. Biotechnol. Biofuels.

[42] Yang L., Chen J., Qin S., Zeng M., Jiang Y., Hu L., Xiao P., Hao W., Hu Z., Lei A. (2018). Growth and Lipid Accumulation by Different Nutrients in the Microalga Chlamydomonas Reinhardtii. Biotechnol. Biofuels.

[43] Breuer G., Evers W.A.C., de Vree J.H., Kleinegris D.M.M., Martens D.E., Wijffels R.H., Lamers P.P. (2013). Analysis of Fatty Acid Content and Composition in Microalgae. J. Vis. Exp..

[44] Kim D.Y., Vijayan D., Praveenkumar R., Han J.I., Lee K., Park J.Y., Chang W.S., Lee J.S., Oh Y.K. (2016). Cell-Wall Disruption and Lipid/Astaxanthin Extraction from Microalgae: Chlorella and Haematococcus. Bioresour. Technol..

[45] Zarinmehr M.J., Daneshvar E., Nigam S., Gopinath K.P.G., Biswas J.K., Kwon E.E., Wang H., Farhadian O., Bhatnagar A. (2022). The effect of solvents polarity and extraction conditions on the microalgal lipids yield, fatty acids profile, and biodiesel properties. Biores. Technol..

[46] Meroiço N.L.C., Leite M.O., Silva C.A.S., Martins M.A., Resende M.E.T., Oliveira E.B., Coimbra J.S.R. (2024). *Tetradesmus obliquus* microalgae: Solvente extraction of lipids under different process conditions. Sci. Agric..

[47] Asevedo E.A., Chagas B.M.E., Júnior S.D.O., Santos E.S. (2023). Recovery of lipids and carotenoids from *Dunaliella salina* microalgae using deep eutectic solvents. Algal Res..

[48] Mienis E., Vandamme D., Foubert I. (2024). Ultrasound assisted extraction of *Nannochloropsis*: Effects on lipid extraction efficiency and lipid stability. Algal Res..

[49] López G., Yate C., Ramos F.A., Cala M.P., Restrepo S., Baena S. (2019). Production of Polyunsaturated Fatty Acids and Lipids from Autotrophic, Mixotrophic and Heterotrophic Cultivation of *Galdieria* sp. strain USBA-GBX-832. Sci. Rep..

[50] Rohit M.V., Mohan S.V. (2018). Quantum Yield and Fatty Acid Profile Variations with Nutritional Mode During Microalgae Cultivation. Front. Bioeng. Biotechnol..

[51] Guihéneuf F., Stengel D.B. (2013). LC-PUFA-Enriched Oil Production by Microalgae: Accumulation of Lipid and Triacylglycerols Containing n-3 LC-PUFA Is Triggered by Nitrogen Limitation and Inorganic Carbon Availability in the Marine Haptophyte Pavlova Lutheri. Mar. Drugs.

[52] da Rosa A.P.C., Moraes L., de Morais E.G., Costa J.A.V. (2020). Fatty Acid Biosynthesis from Chlorella in Autotrophic and Mixotrophic Cultivation. Braz. Arch. Biol. Technol..

[53] Hawrot-Paw M., Ratomski P., Koniuszy A., Golimowski W., Teleszko M., Grygier A. (2021). Fatty Acid Profile of Microalgal Oils as a Criterion for Selection of the Best Feedstock for Biodiesel Production. Energies.

[54] Chen H.H., Jiang J.G. (2017). Lipid Accumulation Mechanisms in Auto- and Heterotrophic Microalgae. J. Agric. Food Chem..

[55] Qin N., Li L., Wang Z., Shi S. (2023). Microbial Production of Odd-Chain Fatty Acids. Biotechnol. Bioeng..

[56] Safi C., Zebib B., Merah O., Pontalier P.Y., Vaca-Garcia C. (2014). Morphology, Composition, Production, Processing and Applications of Chlorella Vulgaris: A Review. Renew. Sustain. Energy Rev..

[57] Hong M.E., Yu B.S., Patel A.K., Choi H.I., Song S., Sung Y.J., Chang W.S., Sim S.J. (2019). Enhanced Biomass and Lipid Production of *Neochloris oleoabundans* under High Light Conditions by Anisotropic Nature of Light-Splitting CaCO_3_ Crystal. Bioresour. Technol..

[58] Hasan F., Shah A.A., Hameed A. (2006). Industrial applications of microbial lipases. Enzym. Microb. Technol..

[59] Peralta-Ruiz Y., González-Delgado A.D., Kafarov V. (2013). Evaluation of Alternatives for Microalgae Oil Extraction Based on Exergy Analysis. Appl. Energy.

[60] Feng K., Kang M., Liu G., Huang Z., Fu J., Wen L., Lan Y., Dai W., Huang Q., Ho C.T. (2023). Effect of Crude Oil Composition and Process Parameters on the Catalytic Performance of Immobilized Lipase during Enzymatic Deacidification of High-Acid Soy Sauce by-Product Oil and Its Bioprocess Scale-Up. LWT.

[61] Nicholson R.A., Marangoni A.G. (2021). Lipase-Catalyzed Glycerolysis Extended to the Conversion of a Variety of Edible Oils into Structural Fats. Curr. Res. Food Sci..

[62] Zheng J., Liang Y., Li J., Lin S., Zhang Q., Zuo K., Zhong N., Xu X. (2023). Enzymatic Preparation of Mono- and Diacylglycerols: A Review. Grain Oil Sci. Technol..

