# Supplementary material for: Integrated Production of Microalgal Oil from Neochloris oleoabundans and Its Enzymatic Conversion into Mono- and Diacylglycerols
Source: Foods. 2026 Jul 1;15(13):2333. doi: 10.3390/foods15132333 (PMC13362220; doi:10.3390/foods15132333)
Supplement: Supplementary file 1 [file foods-15-02333-s001.zip › foods-4374144-supplementary.pdf]

foods

MDPI

Supplementary Material

Table S1: ANOVA tables from the central composite rotatable design (CCRD) applied to autotrophic cultivation conditions.

ANOVA; Var.:Biomass (gL^{-1}); R-sqr=,94865; Adj:,709 (DCCR auto)
4 factors, 1 Blocks, 18 Runs; MS Residual=,0222094
DV: Biomass (gL^{-1})

Factor	SS	df	MS	F	p
**(1)Light intensity (µE m-2 s-1)(L)**	0,001524	1	0,001524	0,06862	0,810318
Light intensity (µE m-2 s-1)(Q)	0,000032	1	0,000032	0,00144	0,972093
(2)Salinity NaCl (gL^{-1})(L)	0,288313	1	0,288313	12,98156	0,036685
Salinity NaCl (gL^{-1})(Q)	0,137299	1	0,137299	6,18201	0,088769
(3)Sodium nitrate (gL^{-1})(L)	0,008636	1	0,008636	0,38883	0,577111
Sodium nitrate (gL^{-1})(Q)	0,009710	1	0,009710	0,43720	0,555722
(4)Sodium Bicarbonate (gL^{-1})(L)	0,043312	1	0,043312	1,95017	0,256954
Sodium Bicarbonate (gL^{-1})(Q)	0,018743	1	0,018743	0,84394	0,426021
1L by 2L	0,018471	1	0,018471	0,83168	0,429028
1L by 3L	0,021095	1	0,021095	0,94980	0,401648
1L by 4L	0,102141	1	0,102141	4,59899	0,121333
2L by 3L	0,107370	1	0,107370	4,83442	0,115309
2L by 4L	0,002147	1	0,002147	0,09666	0,776223
3L by 4L	0,202630	1	0,202630	9,12359	0,056735
Error	0,066628	3	0,022209		
Total SS	1,297472	17			

ANOVA; Var.:Lipid (gL^{-1}); R-sqr=,96766; Adj:,81676 (DCCR auto)
4 factors, 1 Blocks, 18 Runs; MS Residual=,0005375
DV: Lipid (gL^{-1})

Factor	SS	df	MS	F	p
**(1)Light intensity (µE m-2 s-1)(L)**	0,000004	1	0,000004	0,00772	0,935515
Light intensity (µE m-2 s-1)(Q)	0,000563	1	0,000563	1,04652	0,381602
(2)Salinity NaCl (gL^{-1})(L)	0,009288	1	0,009288	17,27820	0,025316
Salinity NaCl (gL^{-1})(Q)	0,005206	1	0,005206	9,68453	0,052799
(3)Sodium nitrate (gL^{-1})(L)	0,001866	1	0,001866	3,47123	0,159335
Sodium nitrate (gL^{-1})(Q)	0,000805	1	0,000805	1,49784	0,308356
(4)Sodium Bicarbonate (gL^{-1})(L)	0,000771	1	0,000771	1,43433	0,317057
Sodium Bicarbonate (gL^{-1})(Q)	0,007783	1	0,007783	14,47862	0,031894
1L by 2L	0,000372	1	0,000372	0,69211	0,466479
1L by 3L	0,009185	1	0,009185	17,08813	0,025689
1L by 4L	0,011402	1	0,011402	21,21144	0,019249
2L by 3L	0,000337	1	0,000337	0,62644	0,486469
2L by 4L	0,001445	1	0,001445	2,68757	0,199665
3L by 4L	0,000682	1	0,000682	1,26817	0,342063
Error	0,001613	3	0,000538		
Total SS	0,049868	17			

Table S2: ANOVA tables from the central composite rotatable design (CCRD) applied to heterotrophic cultivation conditions.

ANOVA; Var.:Biomass (gL^{-1}); R-sqr=,54087; Adj:0, (dccr heterotrofico dia 12)
3 factors, 1 Blocks, 16 Runs; MS Residual=,3911335
DV: Biomass (gL^{-1})

Factor	SS	df	MS	F	p
**(1)Glucose (gL^{-1})(L)**	0,167832	1	0,167832	0,429092	0,536724
Glucose (gL^{-1})(Q)	0,244374	1	0,244374	0,624784	0,459368
(2)Glycerol (gL^{-1})(L)	0,019428	1	0,019428	0,049671	0,831031
Glycerol (gL^{-1})(Q)	1,251388	1	1,251388	3,199390	0,123878
(3)Nitrogen (gL^{-1})(L)	0,089082	1	0,089082	0,227754	0,650080
Nitrogen (gL^{-1})(Q)	0,013903	1	0,013903	0,035545	0,856673
1L by 2L	0,034063	1	0,034063	0,087088	0,777858
1L by 3L	0,011519	1	0,011519	0,029451	0,869383
2L by 3L	0,193904	1	0,193904	0,495748	0,507766
Error	2,346801	6	0,391133		
Total SS	5,111443	15			

Factor	ANOVA; Var.:Lipid (gL^{-1}); R-sqr=,56565; Adj:0, (dccr heterotrofico) 3 factors, 1 Blocks, 16 Runs; MS Residual=,0275364 DV: Lipid (gL^{-1}) SS	df	MS	F	p
**(1)Glucose (gL^{-1})(L)**	0,018965	1	0,018965	0,688724	0,438364
Glucose (gL^{-1})(Q)	0,027478	1	0,027478	0,997889	0,356389
(2)Glycerol (gL^{-1})(L)	0,020629	1	0,020629	0,749153	0,420001
Glycerol (gL^{-1})(Q)	0,014171	1	0,014171	0,514619	0,500108
(3)Nitrogen (gL^{-1})(L)	0,083779	1	0,083779	3,042475	0,131730
Nitrogen (gL^{-1})(Q)	0,003394	1	0,003394	0,123261	0,737524
1L by 2L	0,005417	1	0,005417	0,196727	0,672922
1L by 3L	0,007372	1	0,007372	0,267735	0,623370
2L by 3L	0,001382	1	0,001382	0,050188	0,830170
Error	0,165218	6	0,027536		
Total SS	0,380376	15			

Figure S1: CCRD observed x predicted values for biomass (a) and lipid (b) production under autotrophic conditions.

a)

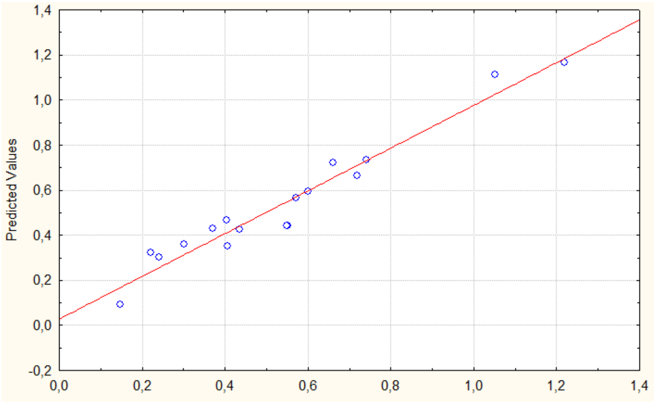

b)

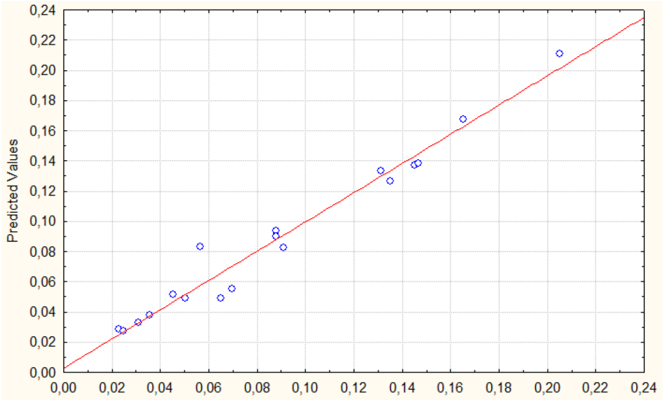

Figure S2: CCRD observed x predicted values for biomass (a) and lipid (b) production under heterotrophic conditions.

a)

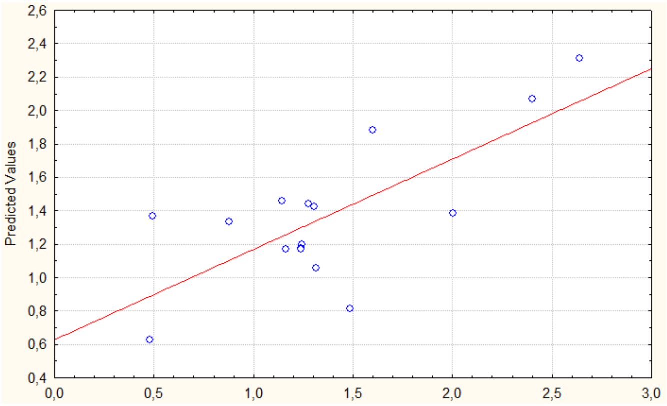

b)

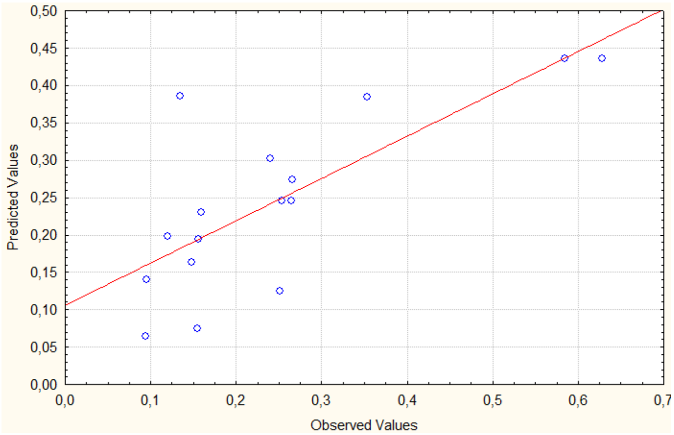
Predicted Values
0,00
0,05
0,10
0,15
0,20
0,25
0,30
0,35
0,40
0,45
0,50
0,0
0,1
0,2
0,3
0,4
0,5
0,6
0,7
Observed Values
